# CircRNA-1926 Promotes the Differentiation of Goat SHF Stem Cells into Hair Follicle Lineage by miR-148a/b-3p/*CDK19* Axis

**DOI:** 10.3390/ani10091552

**Published:** 2020-09-02

**Authors:** Rong H. Yin, Su J. Zhao, Qian Jiao, Ze Y. Wang, Man Bai, Yi X. Fan, Yu B. Zhu, Wen L. Bai

**Affiliations:** 1 College of Animal Science & Veterinary Medicine, Shenyang Agricultural University, Shenyang 110866, China; yinronghuan@163.com (R.H.Y.); jq_name@163.com (Q.J.); wangzeying2012@syau.edu.cn (Z.Y.W.); 2019500012@syau.edu.cn (M.B.); fanyx@syau.edu.cn (Y.X.F.); 2012500032@syau.edu.cn (Y.B.Z.); 2 Sichuan Animal Science Academy, Chengdu 610066, China; zhaosujun0809@163.com; 3 Animal Breeding and Genetics Key Laboratory of Sichuan Province, Chengdu 610066, China

**Keywords:** circular RNA, microRNA, bulge, induced differentiation, post-transcriptional, methylation, cashmere

## Abstract

**Simple Summary:**

Cashmere is the fiber derived from cashmere goats. Its textiles have been favored by consumers due to their typical features, like fine, light, softness, and comfort. Circular RNAs (circRNAs) are thought to play roles in cashmere growth of cashmere goats. CircRNA-1926 was previously identified in cashmere goats, but its functional roles are unclear. In this study, we firstly confirmed the expression of circRNA-1926 in secondary hair follicle bulge of cashmere goats with a significantly higher level at anagen than the counterpart of telogen. Next, we showed that circRNA-1926 promotes the differentiation of hair follicle stem cell into hair follicle lineage in cashmere goats. Mechanistically, we found that circRNA-1926 regulated the *CDK19* expression via sponging miR-148a/b-3p. Our results have demonstrated that circRNA-1926 promotes the differentiation of secondary hair follicle stem cells into hair follicle lineages in cashmere goats through sponging miR-148a/b-3p to promote the expression of the *CDK19* gene. The results from this study provided novel insight into the functional roles of circRNA-1926 in hair follicle regeneration and cashmere growth.

**Abstract:**

Circular RNAs (CircRNAs) are a type of non-coding RNAs, which contain a covalently closed loop structure without 5′ to 3′ free ends. CircRNAs play essential roles in the regeneration of secondary hair follicle (SHF) and cashmere growth in goats. CircRNA-1926 was previously identified in SHF of cashmere goats, but its potential roles are unclear. In this study, we confirmed the expression of circRNA-1926 in SHF bulge of nine cashmere goats with a significantly higher level at anagen than that of telogen. Through the use of both overexpression and siRNA interference, we showed that circRNA-1926 promoted the differentiation of SHF stem cell into hair follicle lineage in cashmere goats which was evaluated via indictor genes Keratin 7 and Keratin 17. Using RNA pull-down, we found that circRNA-1926 bound with miR-148a/b-3p. Additionally, our data indicated that circRNA-1926 promoted the expression of the *CDK19* gene. Using dual-luciferase reporter assays, it was revealed that circRNA-1926 positively regulated the *CDK19* expression through miR-148a/b-3p. The results from this study demonstrated that circRNA-1926 contributes the differentiation of SHF stem cells into hair follicle lineages in cashmere goats via sponging miR-148a/b-3p to enhance *CDK19* expression.

## 1. Introduction

Cashmere is precious natural protein fiber derived from cashmere goats, and its textiles have been favored by consumers due to their typical features, like fine, light, softness, and comfort [1,2]. In cashmere goats, the cashmere growth is controlled by biological cycles of secondary hair follicle (SHF) consisting of anagen, catagen, and telogen [3]. During SHF anagen, the differentiation event of SHF stem cells into hair follicle lineages is closely related to the regeneration and growth of SHF in cashmere goats. The SHF stem cells mainly are found in SHF bulge, and they are a kind of cells with multi-directional differentiation potential and extensive plasticity [4]. During the regeneration and growth of SHF, the stem cells are continuously induced to differentiate into hair follicle lineages under the stimulation of signals from dermal papilla cells, which promotes the formation and growth of cashmere fiber [5].

Several signal pathways and molecules were implicated in regulating the differentiation of hair follicle stem cells into hair follicle lineages, such as Wnt/β-catenin [6], *Lef1* [7], *Lhx2* [8], *TCF3* [9,10], *Wnt10b* [11], and *c-myc* [12]. Additionally, non-coding RNAs might play roles in regulating the differentiation of hair follicle stem cells into hair follicle lineages, like miR-22 [13], and LncRNA-PlncRNA 1 [6]. More recently, circular RNAs (circRNAs) were isolated from skin tissue and SHF of cashmere goats with differential expression level at anagen and telogen, suggesting that they may play functional roles in the reconstruction and growth of cashmere goat SHF [14]. Thus, it was speculated that circRNAs might be involved in regulating the differentiation of hair follicle stem cells into hair follicle lineages in cashmere goats.

The circRNA-1926 was previously identified in cashmere goats that had significantly higher expression level at anagen SHF than the counterpart at telogen [14]. As well known, the differentiation event of hair follicle stem cells into hair follicle lineages is more vigorous at anagen than at telogen [5]. Therefore, we hypothesize that circRNA-1926 may be implicated in the differentiation of SHF stem cells into hair follicle lineages in cashmere goats. Here, we firstly verified the positive expression of circRNA-1926 in SHF bulge of cashmere goats at both anagen and telogen stages. Subsequently, we evaluated the effects of circRNA-1926 on the differentiation of SHF stem cells into hair follicle lineages in cashmere goats via overexpression along with siRNA interference techniques. Further, we explored the potential mechanisms of circRNA-1926 in prompting the differentiation of SHF stem cells into hair follicle lineages. The results from this investigation will provide novel insight into the biological significance of circRNA-1926 in SHF regeneration and cashmere growth in goats. Additionally, our results provide new ideas and scientific basis for artificially regulating the cyclical growth of cashmere to increase cashmere yield and improve cashmere quality in goats.

## 2. Materials and Methods

### 2.1. Skin Tissues of Goats

The experiments were performed following the protocol guidelines approved by Animal Experimental Committee of Shenyang Agricultural University (Shenyang, China) under an ethical code of 201606005. The skin tissue from nine adult female individuals of Liaoning cashmere goats were collected in our previous study [14]. The methods are as follows. Skin tissues of approximately 1 cm^2^ were collected from body side of sampled goats with sterile scalpel blades. The collected skin tissues were firstly washed and sterilized with 75% alcohol. Next, using ophthalmic scissors, the tissue samples were cut into 5 mm^2^ blocks and washed with PBS three times. We treated the sample blocks with 0.25% dispase II at 4 °C overnight. Under a stereomicroscope, the SHFs from each sample were isolated by microseparation in vitro. The isolation of SHF bugle area from each sample was performed according to the described method by Ohyama and Kobayashi [15].

### 2.2. Sequence Analysis of CircRNA-1926

Here, the analyzed circRNA-1926 was previously identified in cashmere goat SHF [16]. The circRNA-1926 is 3887-nt in length, and it exhibited significantly higher level at anagen than the counterpart at telogen [16]. The potential miRNA target sites within circRNA-1926 were predicted and screened through taking intersection of the analysis of three programs: miRDB, RNAhybrid, and miRNA-target, as described in our previous study [1]. We performed the analysis on the nucleotide composition and frequency distribution of nucleotide pairs with MEGA program (Version 6.0) [17].

### 2.3. Cell Cultivation and Overexpression/siRNA Interference Analysis of CircRNA-1926

The SHF stem cells of passage 3 of cashmere goat were utilized for the overexpression/siRNA interference analysis of circRNA-1926. The SHF stem cells were co-cultured with dermal papilla cells (DPCs) for inducing their differentiation into hair follicles lineage, which was performed in transwell devices as described by Yan and colleagues [18]. In brief, the SHF stem cells of passage 3 were plated on six-well plates, and then, a transwell insert was added in which passage 3 DPCs of cashmere goat was seeded. The cells were co-cultured in fresh DMEM/F12 medium (Hyclone, Logan, UT, USA) supplemented with fetal bovine serum of 10% under a humidified atmosphere with 5% CO_2_ at 37 °C. The culture media was changed every two days [19].

In SHF stem cells, the overexpress analysis of circRNA-1926 was conducted via the use of pcDNA3.1 (+) circRNA mini vector (Addgene, Cambridge, MA, USA). The SHF stem cells whose confluence reached 80% were transiently transfected with the recombinant pcDNA3.1 (+) circRNA-1926 or the pMAX-GFP vectors (negative control, Addgene, Cambridge, MA, USA) by Lipofectamine 3000 from Invitrogen (Carlsbad, CA, USA). The SHF stem cells without any treatment were used as blank control cells group, whereas, the SHF stem cells infected with pMAX-GFP (empty vector) were used as negative control group. After transfection 24 h, the overexpression of circRNA-1926 in SHF stem cells was verified by real-time PCR analysis.

Based on the sequence of circRNA-1926 back-spliced junction site, three specific siRNAs were designed and synthesized chemically by GenePharma Co., Ltd. (Shanghai, China), respectively. They were named as Si-circR1 (5′- GGATCATCTTTTTTTTGTCTTC-3), Si-circR2 (5′- CATCTTTTTTTTGTCTTCTTTC-3′), and Si-circR3 (5′- CATATGGATCATCTTTTTTTTG-3′). These three siRNAs have no homology with the goat known genes. For knockdown analysis of circRNA-1926, the siRNAs were transfected into SHF stem cells via siRNAs Lipofectamine RNAiMAX kits (Invitrogen, Shanghai, China).

### 2.4. RNA Pull-Down Assay

The RNA pull-down experiment was conducted as described by He and colleagues [20]. In brief, the biotinylated DNA probe complementary to the black-splice junction sequence of circRNA-1926 (Bio-circRNA-1926-probe: biotin-CATATGGATCATCTTTTTTTTGTCTTCTTTC-biotin) was designed and synthesized by Sangon Biotech Co., Ltd. (Sangon, Shanghai, China). The SHF stem cells were harvested and subjected to cross-linking. Cells were lysed and sonicated. CircRNA-1926 probes were incubated with cell lysates overnight at 37 °C. After hybridization, C1 magnetic beads (Life Technologies, Grand Island, NY, USA), which were subjected to pre cleaning, were added to the lysates, followed by an incubating at 37 °C for 1 h in order to generate the circRNA-1926-probe-beads complex. Finally, the bound RNAs were eluted and extracted by the RNAiso reagent kit (TaKaRa, Dalian, China), and further tested by real-time PCR.

### 2.5. Extraction of Total RNA and Real-Time PCR Reactions

From goat SHF bulges and stem cells, the total RNA was isolated with the RNAiso reagent kit (TaKaRa, Dalian, China). Based on the use of random primers, the first strand cDNA was reversely transcribed by M-MuLV cDNA Synthesis Kit (Sangon, Shanghai, China). One Step PrimeScript microRNA cDNA synthesis kit (TaKaRa, Dalian, China) was used to transcribe the cDNA for the microRNAs testing. We carried out real-time PCR amplification using SYBR Green I assay (TaKaRa, Dalian, China). All primers were designed through the use of Premier Primer 5.0 program (Premier Biosoft International, Palo Alto, CA, USA). The analyzed miRNA mature sequences were obtained from the miRNA database (http://www.mirbase.org, accessed on 28 July 2019), and their sense primers were designed based on the the corresponding miRNA sequence. Whereas, the corresponding anti-sense primers were provided in the kits (TaKaRa, Dalian, China), which are universal reverse primers for all miRNAs analyses. Here, all primers are listed in Supplementary Table S1 with their detailed information. The PCR reaction of each sample was performed in triplicate.

### 2.6. Methylation Detection of CDK19 Gene Promoter in SHF Stem Cells

Based on the use of Methyl Primer Express software (Applied Biosystems, Foster City, CA, USA), we carried out a search for the possible presence of CpG island within a range of 1000-nt of transcription start site upstream of goat *CDK19* gene in goat genome (assembly ARS1, NC_030816.1: 26801290-26972150, https://www.ncbi.nlm.nih.gov/genome/?term=goat, accessed on 28 July 2019). We predicted the potential binding sites of transcription factors within the BSP amplification region (544-nt) by the AliBaba 2.1 program (http://gene-regulation.com/pub/programs.html). From SHF stem cells, the genomic DNA was isolated and treated with MethylCode Bisulfite Conversion Kit (Invitrogen, Shanghai, China). Within the revealed CpG island (680-nt), we designed a pair of primers (BSP-F and BSP-R). A total of 24 CpG sites were included within their potential amplification region. Bisulfite sequencing PCR reactions were performed, and the amplified products were subjected to purification and were cloned into competent *E. coli* DH5α cells. We sequenced 10 positive clones in each group of cells, and the results were displayed using the QUMA program [21].

### 2.7. Dual-Luciferase Reporter Assays

The dual-luciferase reporter assay was conducted as described elsewhere by Yu et al. [22]. In brief, the 3′-untranslated region (3′-UTR) fragment of goat *CDK19* mRNA harboring potential binding sites of miR-148a/b-3p were ligated into pGL3 Basic vector (Promega, Madison, WI, USA). Then, the *CDK19* 3′-UTR fragment reporter vectors were transfected into the SHF stem cells of passage 3 by the Lipofectamine 2000 (Invitrogen, Carlsbad, CA, USA). The transfected cells were cultured under the above-mentioned conditions. After transfection 48 h, the activity of luciferase was examined consecutively via the Dual-Luciferase Reporter Assay System (Promega, Madison, WI, USA).

### 2.8. Statistical Analysis

All obtained data was provided as mean ± SEM. Statistical analyses were performed with SPSS program (Version 17.0). The geometric mean of *UBC*, *YWHAZ*, and *SDHA* was utilized as internal control to normalize the expression of the analyzed genes, which was recommended in a previous publication [23]. Additionally, the geometric mean of chi-let-7d-5p, chi-miR-26a-5p, and chi-miR-15a-5p was utilized as internal control to normalize the expression of analyzed miRNAs, which was recommended in another previous investigation [24]. The means between two groups were analyzed for measuring differences with Student’s *t*-test. The *p* < 0.05 represented the significant difference. Here, all analyzed data was obtained from three replicates.

## 3. Results and Discussion

### 3.1. Sequence Analysis of CircRNA-1926 in Cashmere Goat SHF

**Figure 1:**
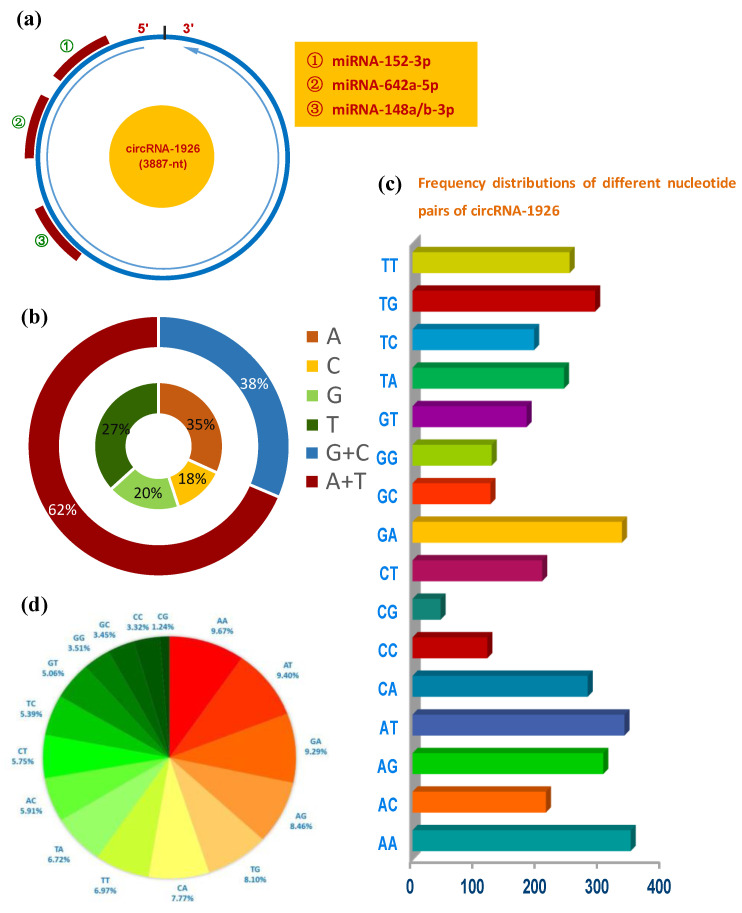
Sequence and structural characteristics of circRNA-1926 in cashmere goats. (**a**) Overall structure of circRNA-1926 with the potential binding sites of multiple miRNAs including miRNA-152-3p, miRNA-642a-5p, and miRNA-148a/b-3p. (**b**) Nucleotide composition of circRNA-1926 and the combined analysis of adenine (A) + thymidine (T), and cytosine (C) + guanine (G). (**c**,**d**) Frequency distributions of different nucleotide pairs of circRNA-1926 in cashmere goats, and their percentage analysis.

The circRNA-1926 is 3887-nt in length that has potential binding sites of four miRNAs: miRNA-152-3p, miRNA-642a-5p, miRNA-148a-3p, and miRNA-148b-3p (Figure 1a). It was revealed that Adenine (A) and Thymine (T) were richer in content than the counterpart of Guanine (G) and Cytosine (C) in circRNA-1926 (Figure 1b), which are highly similar with the reported case on lncRNAs [25]. Additionally, we found that AA, AT, GA, and AG were enriched in circRNA-1926 with the percentages of 9.67%, 9.40%, 9.29%, and 8.46%, respectively (Figure 1c,d). In vivo, although the functional roles of AA- and AT-rich pairs still remain to be further investigated in circRNA molecules, it was found that the AA pair was implicated in the stabilizing size-symmetric RNA internal loops that are important sites for folding and function [26]. Additionally, AT-rich elements in mRNAs have been shown to function as signals for rapid mRNA degradation [27]. Whereas, it was thought that the AG pair-rich in non-coding RNA can pair with GA-rich of its regulatory gene, thereby further to modify the expression of its regulatory gene [28]. On the other hand, it was demonstrated that the GA-pair in RNA is implicated in the structure of loop-loop RNA complex that is essential in trans-activation-responsive RNA [29]. Taken together, it can be speculated that the rich AA, AT, GA, and AG pairs in circRNA-1926 might mean significant function in modulating the expression of their regulatory genes that might be involved in the growth and development of cashmere goat SHF.

### 3.2. Expression Analysis of CircRNA-1926 in Bulge and Its Role in Regulating the Differentiation of SHF Stem Cells into Hair Follicle Lineages

**Figure 2:**
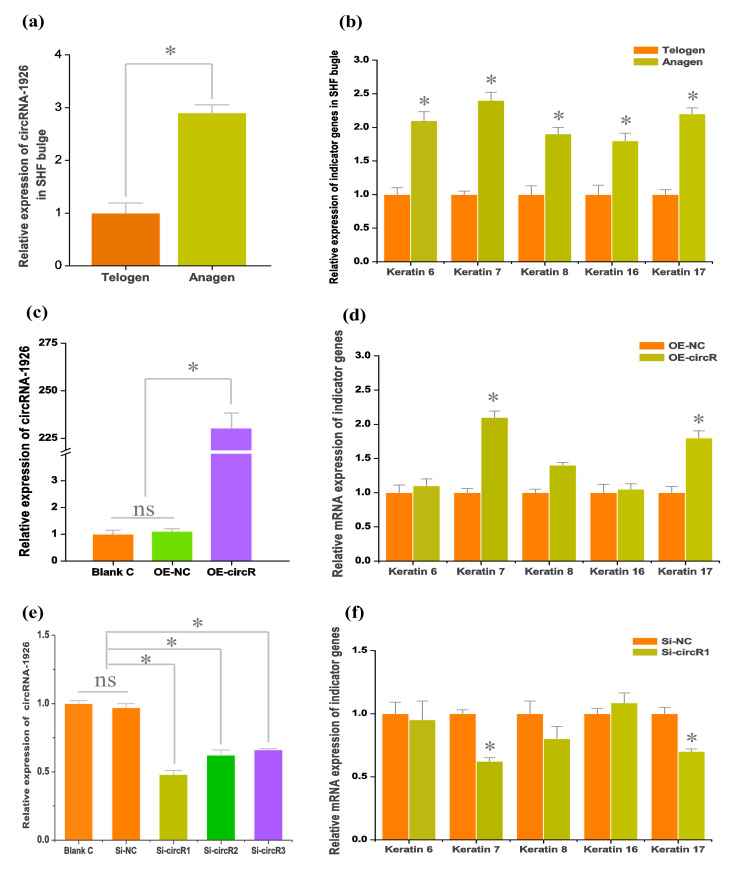
The expression of circRNA-1926 in secondary hair follicle (SHF) bugle of cashmere goats, and its effects on the differentiation of SHF stem cells into hair follicle lineages. (**a**) Expression analysis of circRNA-1926 in SHF bugle of cashmere goats. (**b**) Expression analysis of indicator genes in SHF bugle of cashmere goats. (**c**) Efficiency analysis of circRNA-1926 overexpression in SHF stem cells. (**d**) CircRNA-1926 overexpression significantly promotes the expression of two indictor genes: keratin 7 and keratin 17 in SHF stem cells. (**e**) Efficiency analysis of Si-circR1, Si-circR2, and Si-circR3 in knockdown of circRNA-1926, respectively. (**f**) Si-circRNA1 mediated knockdown of circRNA-1926 significantly decreases the expression of keratin 7 and keratin 17 in SHF stem cells. Blank C = blank cell group, OE-NC = negative control group, and OE-circR = circRNA-1926 overexpression groups. Si-NC = Si-circRNA negative control group. Si-circR1 = Si-circR1 interference group. Si-circR2 = Si-circR2 interference group. Si-circR3 = Si-circR3 interference group. The ns = no significant difference. The asterisk (*) indicates the significant difference (*p* < 0.05).

For revealing the expression pattern of circRNA-1926 in bulge of cashmere goat SHF, two bulge stages were tested including anagen and telogen (Figure 2a). A significantly higher expression of circRNA-1926 at anagen bulge was recorded in comparison to that at telogen. In fact, compared with telogen, the bulge at anagen is under vigorous status during which the bulge stem cells are continuously induced to differentiate into hair follicle lineages under signal stimulation from dermal papilla cells, thereby further to drive the regeneration and development of SHF [30,31]. As shown in Figure 2b, this was also further supported by the obtained results from the present investigation, where several indicator genes were revealed to be significantly higher in expression level at anagen bulge than those of telogen including keratins 6, 7, 8, 16, and 17 [32,33,34]. These observations suggest that circRNA-1926 may be implicated in the differentiation of SHF stem cells into hair follicle lineage in cashmere goats.

To confirm this speculation, circRNA-1926 was overexpressed and silenced in SHF stem cells, respectively. Firstly, the overexpression of circRNA-1926 (OE-circR) increased expression level of circRNA-1926 in SHF stem cells with approximately 230 times which was verified by qPCR assay. Meanwhile, no significant expression difference of circRNA-1926 was recorded between blank control (blank C) group and negative control (OE-NC) group (Figure 2c). Secondly, we investigated the expression changes of several indictor genes in OE-NC and OE-circR groups. We found that the overexpression of circRNA-1926 significantly upregulated the mRNA level of two indictor genes: keratin 7 and keratin 17 (Figure 2d). We also performed siRNA interference experiments of circRNA-1926 in SHF stem cells of cashmere goat by three-independent siRNAs: Si-circR1, Si-circR2, and Si-circR3. We found that the Si-circR1 was more efficient in knockdown of circRNA-1926 in comparison to those of Si-circR2 and Si-circR3 (Figure 2e). Therefore, the Si-circR1 was used in further experiments. As shown in Figure 2f, the knockdown of circRNA-1926 led to a significant decrease in the mRNA expression of keratin 7 and keratin 17 in SHF stem cells in comparison to the counterpart of the OE-NC group (*p* < 0.05, Figure 2f).

In previous investigations, it has been demonstrated that the combinational expression of keratin 7 and keratin 17 is a useful indictor for the lower portion of hair follicle at anagen [32]. Additionally, it was reported that an increasing expression of keratin 17 was recorded around the bulge region and consistently localized to keratinocytes at the advancing front of the emerging hair bulb during the progression of hair follicle anagen [35]. Namely, the increasing expression of keratin 7 and keratin 17 reveals the differentiation of hair follicle stem cells into hair follicular lineage. Thus, taken together with our results, it is implied that circRNA-1926 might have a role in promoting the differentiation of SHF stem cells into hair follicular cells. However, we failed to find any connections among circRNA-1926, miRNAs, and keratins 7/17 by bioinformatics analysis.

### 3.3. CircRNA-1926 Directly Combines with miR-148a/b-3p and May Regulate Their Expression in SHF Stem Cells

**Figure 3:**
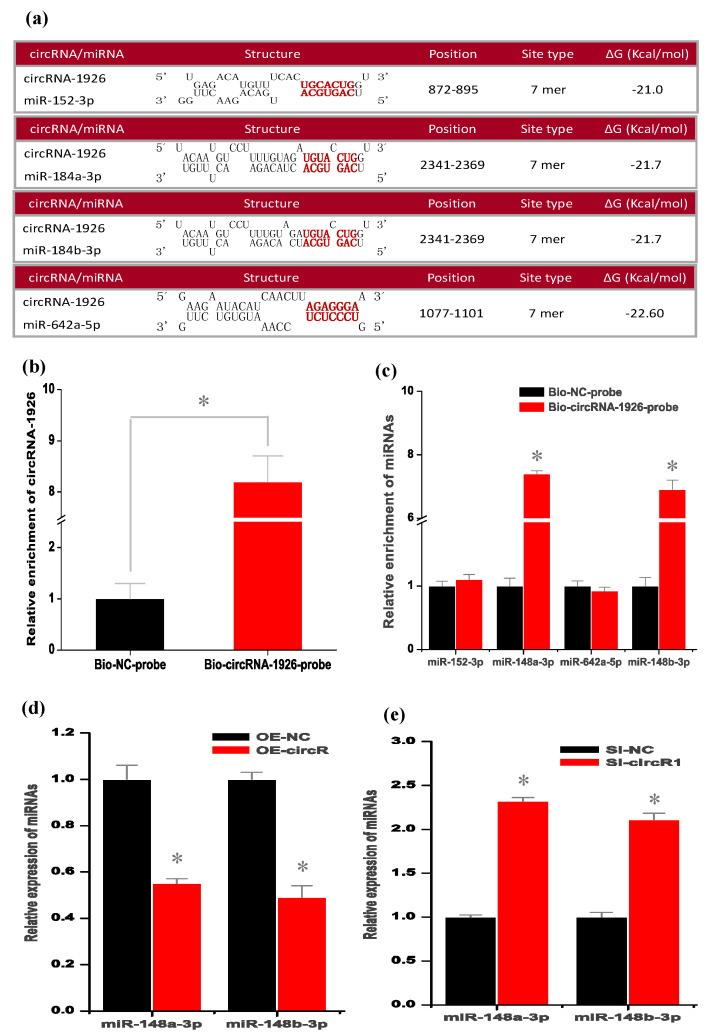
CircRNA-1926 interacts with miR-148a/b-3p and modulates their expression in SHF stem cells. (**a**) The bioinformatics prediction of miRNA potential binding sites on circRNA-1926 sequence. (**b**) Detection results of circRNA-1926 in the same sample pulled down by Bio-circRNA-1926-probe and Bio-NC-probe. (**c**) Detection results of miR-152-3p, miR-184a-3p, miR-184b-3p, and miR-642a-5p in the same sample pulled down by Bio-circRNA-1926-probe and Bio-NC-probe, respectively. (**d**) Detection results of miR-184a-3p and miR-184b-3p in SHF stem cells treated by circRNA-1926 overexpression assay. (**e**) Detection results of miR-184a-3p and miR-184b-3p in SHF stem cells in SHF stem cells treated by Si-circR1 assay. Bio-circRNA-1926-probe = biotinylated circRNA-1926 probe group, Bio-NC-probe = negative control probe group, OE-NC = negative control group, OE-circR = circRNA-1926 overexpression groups. Si-NC = Si-circRNA negative control group. Si-circR1 = Si-circR1 interference group. The asterisk (*) indicates the significant difference (*p* < 0.05).

In several investigations, it was confirmed that circRNAs can serve as “molecular sponge” of miRNAs to prevent their binding to corresponding target mRNAs [36,37,38,39]. Here, based on bioinformatics prediction, we showed that circRNA-1926 contained potential binding sites of four miRNAs, including miR-152-3p, miR-184a-3p, miR-184b-3p, and miR-642a-5p (Figure 3a). Among them, to determine which miRNAs could combine with circRNA-1926, we performed RNA pull-down analysis through biotinylated DNA probes complementary to the black-splice junction sequences of circRNA-1926. As observed in Figure 3b, in the Bio-circRNA-1926-probe (circRNA-1926 probe) pulled down pellet, we verified the higher enrichment of circRNA-1926 in comparison to that of the Bio-NC-probe (negative control probe). Interestingly, we noted that the Bio-circRNA-1926-probe (circRNA-1926 probe) pulled down pellet also had higher enrichment of miR-148a-3p and miR-148b-3p, but not for miR-miR-152-3p and miR-642a-5p, in comparison to that of Bio-NC-probe (Figure 3c). Thus, it can be inferred that circRNA-1926 might interact directly with both miR-148a-3p and miR-148b-3p via acting as their “molecular sponge”.

To define whether circRNA-1926 regulates the expression of miR-148a/b-3p, we performed the overexpression of circRNA-1926 in SHF stem cells by AdEasy Adenoviral vector assay. As observed in Figure 3d, the overexpression of circRNA-1926 significantly downregulated the expression of miR-148a/b-3p in SHF stem cells in comparison to the negative control (*p* < 0.05). On the contrary, the Si-circR1 mediated knockdown of circRNA-1926 significantly upregulated the expression of miR-148a/b-3p (Figure 3e). However, we found that the expression of circRNA-1926 was not significantly changed in SHF stem cells after overexpression or knockdown of miR-148a/b-3p, respectively (data not shown). Thus, it appears to become apparent that circRNA-1926 may regulate the expression of miR-148a/b-3p in SHF stem cells. More recently, in an investigation on colorectal carcinoma invasion and metastasis, Han and colleagues (2020) reported a highly similar regulatory pattern where the authors confirmed that circLONP2 directly interacted with miR-17 and regulated its expression, thereby to enhance [40]. On the other hand, several functional roles have been identified for circRNAs, such as serving as miRNA sponges [37,41], protein binding [42], RNA transport [43], regulation of gene transcription and protein translation [44,45]. In this study, we showed that circRNA-1926 directly interacted with both miR-148a/b-3p (Figure 3d), and negatively regulated their expression in SHF stem cells (Figure 3e). However, the other potential functional roles of circRNA-1926 in SHF stem cells should be further researched such as its possible effects on the renewal, activation, and proliferation of SHF stem cells.

### 3.4. CircRNA-1926 Promotes CDK19 Expression without Modulating the Methylation Level of Its Promoter Region

**Figure 4:**
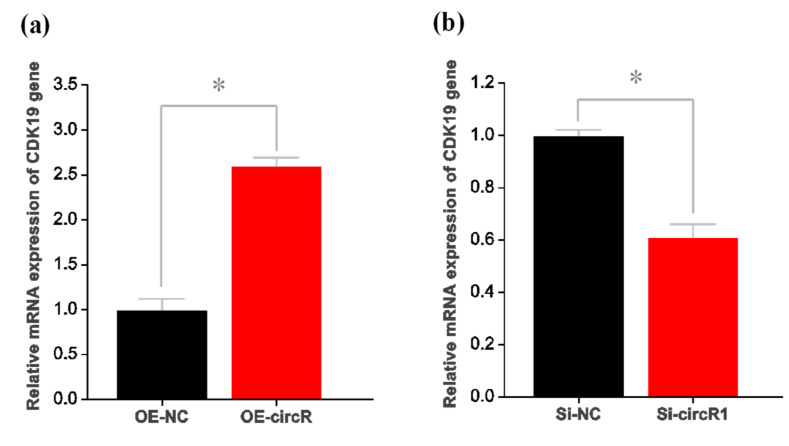

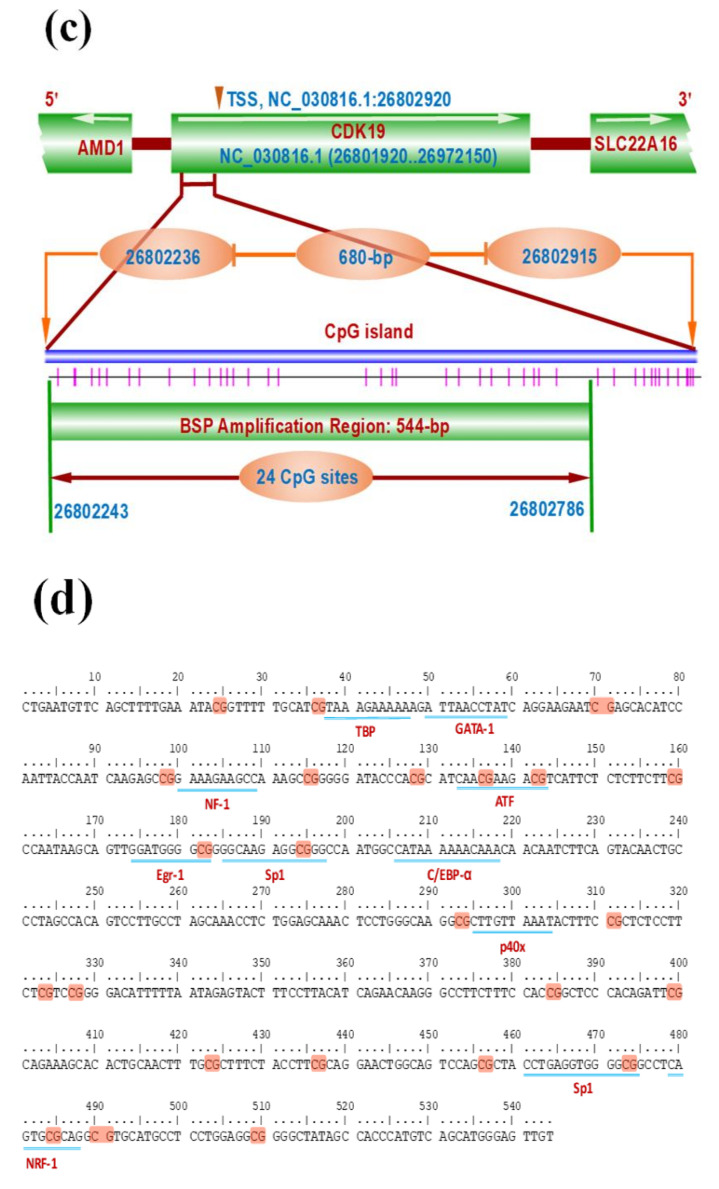

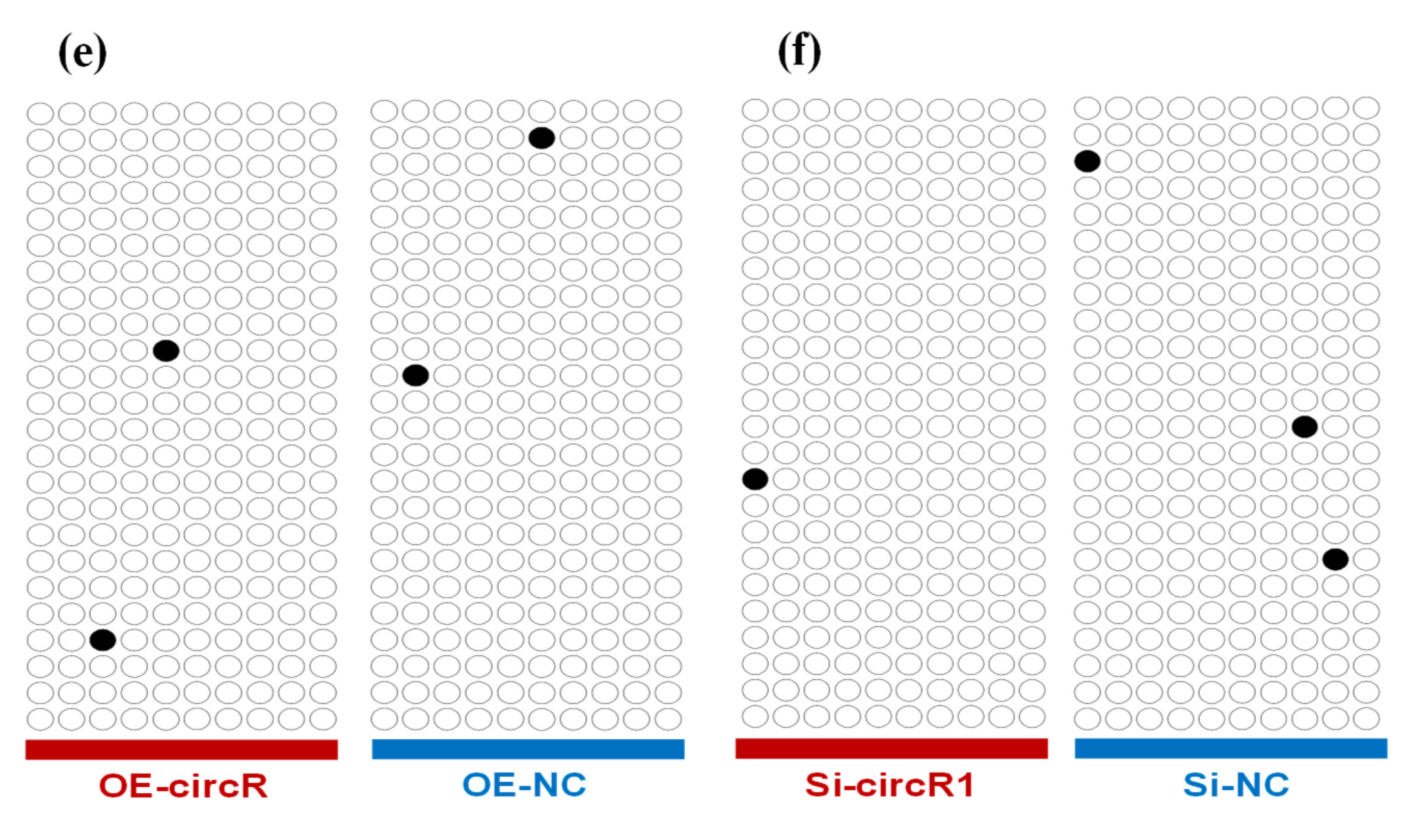
CircRNA-1926 promotes the expression of *CDK19* in SHF stem cells without changing its promoter methylation. (**a**) Analysis results of *CDK19* mRNA in SHF stem cells treated by circRNA-1926 overexpression assay. (**b**) Analysis results of *CDK19* mRNA in SHF stem cells treated by silnR-1 assay. The asterisk (*) indicates the significant difference (*p* < 0.05). (**c**) A graph of CpG islands in the promoter region of the *CDK19* gene where the CpG sites are indicated by short pink vertical lines. The nucleotide positions in the *CDK19* gene are determined based on the sequence NC_030816.1 in goat genome (https://www.ncbi.nlm.nih.gov/genome/?term=goat). BSP = bisulfite sequencing PCR. (**d**) Transcription factors having potential binding sites within the BSP amplification region of *CDK19* gene promoter. The CpG sites are marked with orange shadow regions. The potential binding sites of transcription factors are marked by blue underlined line along with their corresponding name. (**e**,**f**) The BSP results of *CDK19* promoter in SHF stem cells treated with circRNA-1926 overexpression or knockdown assay. Filled black circles and unfilled white circles represented the methylated and unmethylated CpG sites, respectively. OE-NC = negative control group, OE-circR = circRNA-1926 overexpression groups, Si-NC = Si-circRNA negative control group, and Si-circR1 = Si-circR1 interference group.

To determine the underlying mechanisms of circRNA-1926 in regulating the differentiation of SHF stem cells into hair follicle lineages, we further investigated the expression changes of several potential target genes of miR-148a/b-3p in SHF stem cells with overexpression or knockdown of circRNA-1926. Here, the potential target genes of miR-148a/b-3p were bioinformatically predicted in our previous study including *CDK19*, *SOS2*, *RPS6KA5*, *HOMER1*, *WDR47*, *LDLR*, *BCL2L11*, *UBE2D3*, *NPTN*, *MEOX2*, *CBLB*, and *KMT2A* [14]. As a result, interestingly, only the expression of *CDK19* was significantly changed after overexpression or knockdown of circRNA-1926 in SHF stem cells (Figure 4a,b). Thus, it can be inferred that circRNA-1926 might be positively involved in *CDK19* expression in SHF stem cells via certain mechanisms.

It is known that promoter methylation is widely implicated in regulating gene transcriptional level without the alteration of DNA sequence [46,47]. This draws us to ask whether circRNA-1926 modifies the methylation degree within the promoter region of the *CDK19* gene, thereby to regulate its transcriptional expression in SHF stem cells of cashmere goats. Correspondingly, we investigated the methylation changes within the *CDK19* gene promoter in SHF stem cells with overexpression or knockdown of circRNA-1926. A CpG island of 680-bp was revealed in the promoter region of goat *CDK19* gene (Figure 4c). A fragment of 544-bp was amplified which harbored 24 CpG sites (Figure 4d) and several transcriptional factor (TF) binding sites, like *TBP*, *GATA-1*, *NF-1*, *ATF*, *Egr-1*, and *Sp1* (Figure 4d). We displayed the obtained results on methylation analysis of the *CDK19* gene promoter region in SHF stem cells with overexpression or knockdown of circRNA-1926 (Figure 4e,f). The methylation degree within the promoter region of *CDK19* gene appears to be not involved in the modulation of circRNA-1926 on the expression of the *CDK19* gene in SHF stem cells.

Additionally, it is widely accepted that CpG islands are generally unmethylated, but they may become methylated under few cell-type-specific conditions. Moreover, a regulatory mechanism was recognized that circRNA may modulate the promoter methylation degree of its regulatory gene, thereby further to regulate its transcriptional expression [48]. For example, it was reported that circRNA-5692 decreased the methylation levels of the promoter region of the *DAB2IP* gene, thereby to further upregulate the expression level of the *DAB2IP* gene [49]. Thus, in this study, we also investigated the methylation status of *CDK19* gene promoter region in SHF stem cells with overexpression or knockdown of circRNA-1926 for revealing the possible methylation events.

### 3.5. CircRNA-1926 Positively Regulates the Expression of CDK19 through miR-148a/b-3p

**Figure 5:**
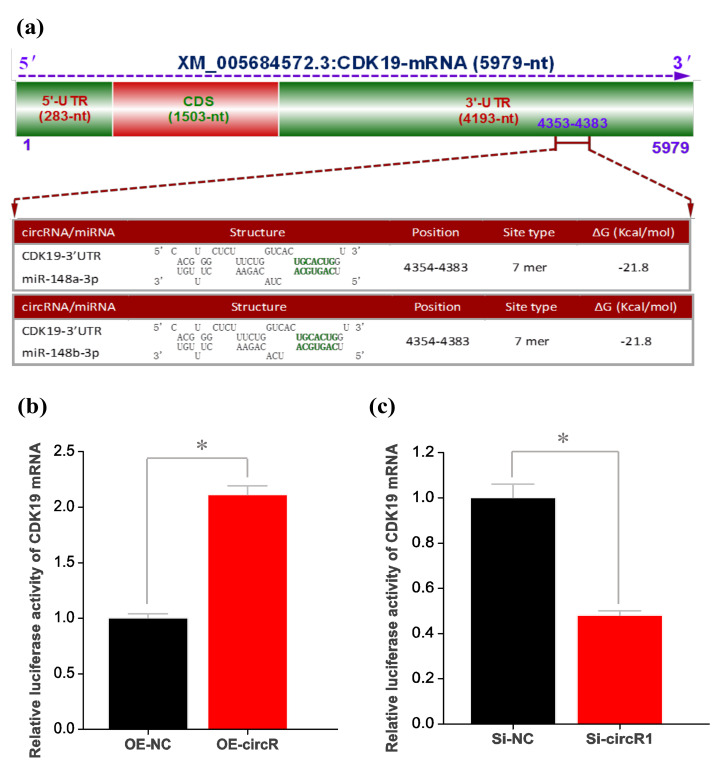
The effect of miR-148a/b-3p on *CDK19* expression in SHF stem cells. (**a**) A structural graph of goat *CDK19* mRNA and the analysis on potential binding sites of miR-148a/b-3p within *CDK19* mRNA 3′-UTR. (**b**) Relative luciferase activities of *CDK19* mRNA 3′-UTR reporter in SHF stem cells transfected with OE-NC or OE-circR. (**c**) Relative luciferase activities of *CDK19* mRNA 3′-UTR reporter in SHF stem cells transfected with Si-NC or Si-circR1. OE-NC = negative control group, OE-circR = circRNA-1926 overexpression groups, Si-NC = Si-circRNA negative control group, and Si-circR1 = Si-circR1 interference group. The asterisk (*) indicates the significant difference (*p* < 0.05).

Several investigations revealed that circRNA may modulate the expression of target gene through serving as miRNA “molecular sponge” to disinhibit its target mRNA [50,51,52]. Here, we confirmed that circRNA-1926 directly interacted with miR-148a/b-3p (Figure 3c). This promotes us to ask whether the revealed positive regulation of circRNA-1926 on the expression of the *CDK19* gene may be achieved via miR-148a/b-3p mediated regulation. Thus, we carried out a prediction for potential binding sites of miR-148a/b-3p within the 3′-UTR region of *CDK19* mRNA. As a result, interestingly, a potential binding site of miR-148a/b-3p was harbored in the 3′-UTR region of *CDK19* mRNA (Figure 5a). In order to verify the predicted interaction between 3′-UTR region of *CDK19* mRNA and miR-148a/b-3p, dual-luciferase reporter assay was conducted in SHF stem cells. We used the reporter vector of goat *CDK19* mRNA 3′-UTR that harbored the potential binding site for miR-148a/b-3p. As shown in Figure 5b, the overexpression of circRNA-1926 (OE-circR) in SHF stem cells significantly increased the relative luciferase activity of *CDK19* mRNA 3′-UTR in comparison to negative control (OE-NC). Whereas, the Si-circR1 mediated knockdown of cicRNA-1926 (Si-circR1) in SHF stem cells significantly decreased the relative luciferase activity of *CDK19* mRNA 3′-UTR compared with negative control (Si-NC) (Figure 5c). Taken together with above results, a molecular mechanism was inferred that circRNA-1926 regulated the CDK19 expression in SHF stem cells via sponging miR-148a/b-3p. The *CDK19* is previously also known as *CDK8*-like and *CDK8L* because of its high amino acid conservation with *CDK8* [53]. Although it is not yet known whether *CDK19* plays different physiological roles with *CDK8* in the functional activity of Wnt/β-catenin signaling, it was demonstrated that *CDK8* was required in the activation of Wnt/β-catenin signaling [54]. Whereas, it is known that the Wnt/β-catenin signaling plays important roles in the differentiation of hair follicle stem cells into hair follicle lineages [6,55,56,57]. Therefore, we speculate that *CDK19*, as a highly conserved paralog of *CDK8*, may be also implicated in the Wnt/β-catenin signaling. Taken together, it can be inferred that circRNA-1926 sponged miR-148a/b-3p to promote the differentiation of SHF stem cells into hair follicle lineages through positively regulating the expression of the *CDK19* gene in cashmere goats.

## 4. Conclusions

We showed that circRNA-1926 promotes the differentiation of SHF stem cells into hair follicle lineages in cashmere goats by regulating the miR-148a/b-3p/*CDK19* axis.

## Supplementary Materials

The following are available online at https://www.mdpi.com/2076-2615/10/9/1552/s1, Figure S1: A protein sequence alignment between *CDK8* and *CDK19* in goat, Table S1: Details of the primers in this study and their corresponding amplicon size.
