# Supplementary material for: CircRNA-1926 Promotes the Differentiation of Goat SHF Stem Cells into Hair Follicle Lineage by miR-148a/b-3p/CDK19 Axis
Source: Animals (Basel). 2020 Sep 2;10(9):1552. doi: 10.3390/ani10091552 (PMC7552268; doi:10.3390/ani10091552)
Supplement: Supplementary file 1 [file animals-10-01552-s001.pdf]

```
goat CDK8 (XP_005687614.1)   MDYDFKVKLSSERERVEDLFEYEGCKVGRGTYGHVYKAKRKDGKDDKDYALKQIEGTGIS 60
goat CDK19 (XP_017908559.1)  ------------------------------------------------------------ 1

goat CDK8 (XP_005687614.1)   MSACREIALLRELKHPNVISLQKVFLSHADRKVWLLFDYAEHDLWHIIKFHRASKANKKP 120
goat CDK19 (XP_017908559.1)  ...................A........S............................... 60

goat CDK8 (XP_005687614.1)   VQLPRGMVKSLLYQILDGIHYLHANWVLHRDLKPANILVMGEGPERGRVKIADMGFARLF 180
goat CDK19 (XP_017908559.1)  M....S...................................................... 120

goat CDK8 (XP_005687614.1)   NSPLKPLADLDPVVVTFWYRAPELLLGARHYTKAIDIWAIGCIFAELLTSEPIFHCRQED 240
goat CDK19 (XP_017908559.1)  ............................................................ 180

goat CDK8 (XP_005687614.1)   IKTSNPYHHDQLDRIFNVMGFPADKDWEDIKKMPEHSTLMKDFRRNTYTNCSLIKYMEKH 300
goat CDK19 (XP_017908559.1)  ......F.........S.............R....YP..Q.....T..A.S......... 240

goat CDK8 (XP_005687614.1)   KVKPDSKAFHLLQKLLTMDPIKRITSEQAMQDPYFLEDPLPTSDVFAGCQIPYPKREFLT 360
goat CDK19 (XP_017908559.1)  .......V.L..........T........L.....Q......L................N 300

goat CDK8 (XP_005687614.1)   EEEPDDKGDKKNQQQQ-----------------------PGNNHTNGTG-----HPG--- 389
goat CDK19 (XP_017908559.1)  .D..EE....NQ....NQHQQPTAPPQQAAAPPQAPPQQQNSTQ....AGGAGAG.AGAG 360

goat CDK8 (XP_005687614.1)   ---NQDSSHTQGPPLKKVRVVPPTTTSGGLIMTSDYQRSNPHAAYPNPGPSTSQPQSSMG 446
goat CDK19 (XP_017908559.1)  LQHS...GLN.V..N..P.LG.SG.N...PV.P....H.SSRLN.QSSVQGS..S..TL. 420

goat CDK8 (XP_005687614.1)   YSTTSQQPPQY--SHQTHRY 464
goat CDK19 (XP_017908559.1)  ..SS...SA..HP...A... 440
```

Figure S1. A protein sequence alignment between CDK8 and CDK19 in goat.

Table S1. Details of the primers in this study and their corresponding amplicon size.

Genes	Reference	Primer pair with sequence (5′–3′)[a]	Primer length (nt)	TA[b] (℃)	Amplicon size[c] (bp)
circRNA-1926	[16]	F: GGTGGCATCGATAATCCTGC	20	55	182
		R: ACAGATCCCACATTTCAGAACT	22		
Keratin 6	XM_018049019.1 in GenBank	F: CAGTCGCAGCCTCTACAACCT	21	56	159
		R: CAAATGCCACCTCCATAACCA	21		
Keratin 7	XM_005680100.3 in GenBank	F: GAGTTTGTGGTGTTGAAGAA	20	56	194
		R: AAGTCCAGGGAGCGGTTGTT	20		
Keratin 8	XM_005679931.3 in GenBank	F: TCCTTCAGCAGCCGCTCCTA	20	58	160
		R: CTGTAATGCCCCCCAAACCT	20		
Keratin 16	XM_013972083.2 in GenBank	F: CCTTTGTGGCTAGTGGTATG	20	55	188
		R: CAGTTTCAGGGGTTGCTTAT	20		
Keratin 17	XM_018065055.1 in GenBank	F: GGGGAATGGAAACAGAGGAG	20	56	112
		R: GAGGAGAGAAGCCCAAGATG	20		
CDK19	XM_005684572.3 in GenBank	F: TGAGAGGGGGAGAGTCAAAATA	22	55	104
		R: ACCAGAAAGTCACAACTACAGG	22		
UBC	[23]	F:GCATTGTTGGGTTCCTGTGT	20	52	90
		R:TTTGCATTTTGACCTGTGAG	20		
YWHAZ	[23]	F:TGTAGGAGCCCGTAGGTCATCT	22	56	102
		R:TTCTCTCTGTATTCTCGAGCCATCT	25		
SDHA	[23]	F:AGCACTGGAGGAAGCACAC	19	53	105
		R:CACAGTCGGTCTCGTTCAA	19		
CDK19[d]	The present study	F:TTGAATGTTTAGTTTTTGAAAT	22	55	544
		R:ACAACTCCCATACTAACATAAA	22		
miR-152-3p	MIMAT0000162 in miRBase	F:CGTCAGTGCATGACAGAACTTGG	23	62	N.A.
miR-148a-3p	MIMAT0035977 in miRBase	F:CGTCAGTGCACTACAGAACTTTGT	24	60	N.A.
miR-148b-3p	MIMAT0035979 in miRBase	F:CGTCAGTGCATCACAGAACTTTGT	24	62	N.A.
miR-642a-5p	MIMAT0003312 in miRBase	F:CGGTCCCTCTCCAAATGTGTCTTG	24	63	N.A.
let-7d-5p	[24]	F:CGAGAGGTAGTAGGTTGCATAGTT	24	62	N.A.
miR-26a-5p	[24]	F:CGTTCAAGTAATCCAGGATAGGCT	24	61	N.A.
miR-15a-5p	[24]	F: CGTAGCAGCACATAATGGTTTGTG	24	63	N.A.

[a] F: forward, R:reverse; [b] TA annealing temperature; [c] N.A.: not available; [d] CDK19 was used for the designing of BSP primers.
